# Recent Advances in Porphyrin-Based COFs Boosting CO_2_ Photocatalytic and Electrocatalytic Conversion

**DOI:** 10.3390/nano15231787

**Published:** 2025-11-27

**Authors:** Jiatong Yin, Linxue Sang, Yue Wang

**Affiliations:** School of Chemistry and Chemical Engineering, Jiangsu University, Zhenjiang 212013, China; 2222312013@stmail.ujs.edu.cn (J.Y.); 2222312076@stmail.ujs.edu.cn (L.S.)

**Keywords:** porphyrin, covalent organic frameworks (COFs), synthesis, carbon dioxide conversion, recent advances

## Abstract

Porphyrins are conjugated tetrapyrrolic macrocycles with tunable photophysical and catalytic properties, while covalent organic frameworks (COFs) are crystalline, porous polymers built from robust covalent linkages. Combining these motifs yields porphyrin-based COFs that couple ordered porosity with light-harvesting and metal-anchoring capabilities, offering promise for carbon dioxide capture and conversion. This review provides an integrated overview of their design, synthesis, structure, and function in the context of CO_2_ capture, storage, and photocatalytic/electrocatalytic reduction. We survey recent literature, organize materials by linkage chemistry and topology, and summarize metallation, peripheral functionalization, and heterostructure strategies, compiling representative performance metrics where reported. The collected studies indicate that appropriate metallation and π-extension enhance light absorption and charge separation; high crystallinity and accessible pores facilitate mass transport; and electronic coupling to conductive phases improves catalytic activity and selectivity in CO_2_ reduction. We close by outlining challenges and opportunities, including improving charge transport without sacrificing stability, pinpointing and quantifying active sites, and operando characterization to connect structure with function. This objective synthesis is intended to guide rational design of porphyrin-COFs for efficient and durable CO_2_ management.

## 1. Introduction

Porphyrin, historically known as the “purple pigment”, is an essential chromophore in living systems. Porphyrins and their derivatives are widespread in biology and occur in key organelles involved in energy transfer. The porphyrin macrocycle contains 18 π electrons; as a prototypical conjugated tetrapyrrolic ring, it offers exceptional tunability through substitutions at the peripheral *β*- and *meso*-positions, modification of the inner core, and diverse linkage modes [1]. Owing to these features, porphyrins find broad applications in sensing, energy conversion, and biomedicine [2,3,4,5,6,7,8,9,10,11].

Covalent organic frameworks (COFs) are a rapidly developing class of crystalline porous polymers in which light-element building blocks are linked by covalent bonds into two- or three-dimensional periodic π-array networks with intrinsic porosity. COFs show great promise in gas adsorption, heterogeneous catalysis, sensing, and energy conversion [10,12]. When porphyrins are used as structural units, the resulting porphyrin-based COFs constitute a family of highly ordered, permanently porous, and robust organic frameworks. Benefiting from extended π-electron delocalization and high specific surface areas, porphyrin COFs (Por-COFs) have been widely explored for optoelectronics, molecular electronics, separations, therapy, photosynthesis, energy conversion and catalysis [9,13,14,15,16,17,18,19,20].

Porphyrin-based COFs merge the molecular precision of porphyrins with the long-range order and permanent porosity of COFs, enabling simultaneous control over light harvesting, mass transport, and site isolation within one scaffold. Such integration mitigates fast charge recombination typical of molecular porphyrins while preserving tunable coordination environments at the M–N_4_ centers, a combination that is advantageous for CO_2_ capture and conversion. In the porphyrin COFs surveyed in this review, these M–N_4_ sites are introduced in almost all cases prior to framework construction by employing pre-metallated porphyrin building blocks, whereas post-synthetic metalation of a pre-formed porphyrin COF is much less common. Key levers include linkage chemistry (from imine to sp^2^-carbon), topology and stacking control, and electronic coupling to conductive phases, each of which impacts carrier separation, diffusion pathways, and product selectivity in CO_2_ reduction.

Recent studies illustrate how structural design translates into function. Undulating (non-planar) 2D architectures attenuate interlayer π–π stacking, expose buried active sites, and facilitate mass transfer, thereby improving photocatalytic CO_2_RR; in representative systems, optimizing steric and electronic effects around Co–porphyrin nodes markedly enhanced activity and CO selectivity [21]. Beyond 2D sheets, three-dimensional frameworks expand accessible topologies and hierarchical pores for faster substrate/product diffusion and diversified confinement environments, offering complementary pathways to engineer charge transport and catalytic microenvironments [22].

In parallel, heterostructure and host–guest strategies further enrich the design space. Embedding porphyrinic nanodomains (e.g., metalloporphyrin-derived carbon dots) within COF channels combines the advantages of homogeneous and heterogeneous catalysis while suppressing leaching, yielding stable, selective CO formation under visible light. These examples underscore that rationally coupling active-site chemistry with meso-/nano-scale transport engineering is central to performance gains [23].

Against this backdrop, this review organizes porphyrin-COFs by linkage and dimensionality, distills structure–property–activity relationships across photo/electrocatalytic CO_2_RR, and highlights design rules spanning metallation, π-extension, pore/stacking regulation, particle-size control, and interfacial coupling. We close by outlining persistent challenges—quantifying operando active states, reconciling conductivity with stability, and steering C1→C2^+^ selectivity, along with emerging opportunities in tandem catalysis and scalable synthesis.

## 2. Structure and Properties of Porphyrin-Based COFs

Covalent organic frameworks (COFs) are a rapidly advancing class of porous organic crystalline materials in which building blocks are covalently linked. Their intrinsic porosity, high crystallinity, in-plane π-conjugated sheets, and ordered interlayer π–π columnar stacking endow them with outstanding physicochemical properties [12]. Framework topology—particularly square-lattice (sql) connectivity—plays a decisive role in defining pore architecture. Leveraging the near-square, planar geometry of porphyrins as four-connecting nodes, porphyrin-based COFs offer greater design flexibility than COFs constructed from many other linkers.

From a property viewpoint, these structural motifs translate into characteristic optical, electronic, and stability features that are highly relevant to CO_2_ conversion. Extended in-plane π-conjugation and columnar π–π stacking give rise to intense Soret and Q bands that are often red-shifted and broadened relative to the molecular porphyrins, reflecting exciton delocalization and enhanced light harvesting across the visible region. Metallation at the porphyrin N_4_ core and π-extension through conjugated linkers further modulate the frontier orbital energies, band gaps, and intersystem-crossing efficiencies, thereby tuning charge-separation lifetimes and redox windows for CO_2_ reduction. On the electronic side, densely packed π-columns in 2D architectures support anisotropic charge transport, whereas 3D porphyrin COFs with interconnected cages and channels can provide more isotropic pathways and shortened diffusion lengths for ions and molecules. The same structural elements also govern stability: more robust linkages such as β-ketoenamine, triazine, or fully sp^2^-carbon-connected backbones generally afford superior hydrolytic and thermal stability than boronate esters or simple imines, and the chelating M–N_4_ coordination environment helps to suppress metal leaching under operating conditions. Thus, the interplay between topology, linkage chemistry, stacking, and metallation establishes a direct structure–property–activity relationship that underpins the porphyrin COF platforms discussed in the following sections.

**Figure 1:**
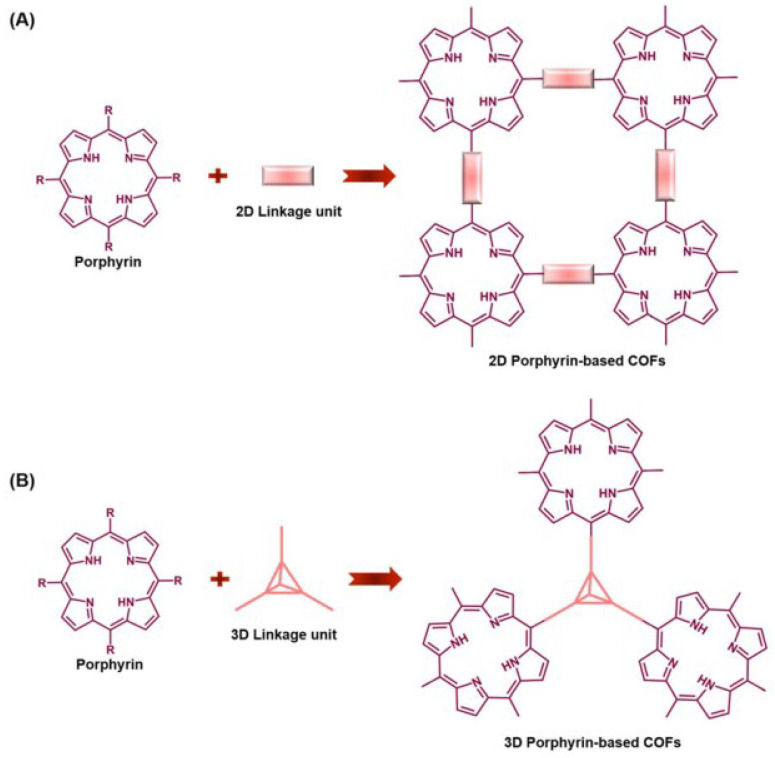
Synthesis strategy for the assembly of porphyrin-based COFs: (**A**) 2D Porphyrin-based COFs; (**B**) 3D Porphyrin-based COFs. Reproduced from [24], with permission from Royal Society of Chemistry, 2024.

By topology and dimensionality, COFs are generally categorized as two-dimensional (2D) or three-dimensional (3D) networks (Figure 1) [24]. In 2D COFs, monomers are covalently connected within a plane to form layered structures; adjacent layers stack via π–π interactions into columnar arrays, creating one-dimensional channels whose size and shape depend sensitively on the stacking registry. In 3D COFs, monomers extend covalently along all three spatial directions to yield periodic frameworks [25]. Unlike their 2D counterparts, 3D COFs commonly develop large cage-like cavities and frequently exhibit multi-fold interpenetration as growth proceeds outward from reactions occurring within the expanding cavities [13,25].

### 2.1. Two-Dimensional Porphyrin COFs

From a structural standpoint, the defining advantage of two-dimensional COFs is their ability to assemble into periodic, columnar π–π arrays [25]. Such architectures give rise to long-lived photoexcited states, preorganized charge-transport pathways, and high charge-carrier mobilities, making two-dimensional (2D) COFs highly effective heterogeneous photocatalysts. Moreover, crystallinity is crucial for establishing clear structure–property–activity relationships and thereby improving photocatalytic efficiency [26].

**Figure 2:**
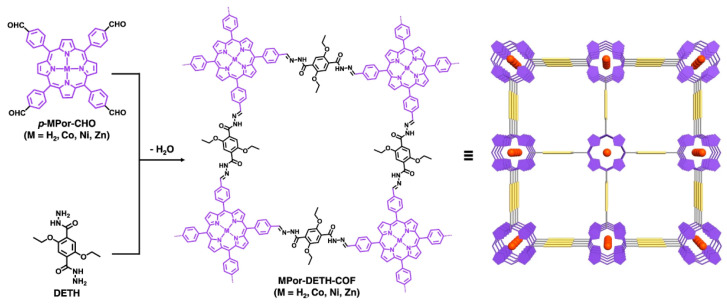
Chemical structure. Schematic representation of the synthesis of MPor-DETH-COFs in a mixed solvent of 1,2-dichlorobenzene, *n*-butanol, and aqueous acetic acid at 120 °C. Reproduced from [25], with permission from Springer Nature, 2021.

Wang and co-workers [25] reported the synthesis and characterization of four isostructural, hydrazone-linked 2D porphyrin COFs (Figure 2). Aldehyde-functionalized porphyrins *p*-MPor-CHO (M = H_2_, Co, Ni, Zn) were condensed with the dihydrazide linker DETH to afford MPor-DETH-COFs (M = H_2_, Co, Ni, Zn) with high crystallinity and large specific surface areas. The powder X-ray diffraction (PXRD) experiment was performed to elucidate the crystalline nature of these COFs. Notably, incorporation of different transition-metal ions within the porphyrin ring markedly modulated the carrier dynamics of the resulting frameworks.

**Figure 3:**
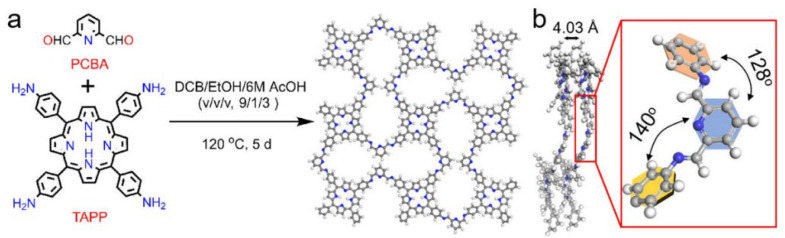
Structure of COF-366: (**a**) Schematic representation of the synthesis procedure and structure; (**b**) Side view of the structure (the red frame is enlarged image of the circled three-dimensional molecular structure. Reproduced from [27], with permission from John Wiley and Sons, 2022.

Lan and colleagues [27] designed a non-linear network in which 2,6-pyridinedicarboxylic acid (PCBA) bridges 5,10,15,20-tetrakis(4-aminophenyl)porphyrin (TAPP) to form H_2_-TPCOF (Figure 3). The crystal structure of H2-TPCOF was elucidated by experimental PXRD measurements along with theoretical structural simulations by using the Materials Studio 7.0 software. The post-metalation yielded M-TPCOFs (M = Co, Cu) with markedly enhanced sensing performance; in particular, Co-TPCOF exhibited high selectivity for NO_2_ and a sensitivity among the highest reported for 2D materials and COFs.

Determining the stacking arrangement in 2D covalent organic frameworks (COFs), such as AA or AB stacking, is a multifaceted challenge with significant implications for their structure–property relationships, including charge transport, optical behavior, and mechanical stability. Accurate identification often relies on a combination of advanced powder X-ray diffraction (PXRD) techniques, high-resolution transmission electron microscopy (TEM), and computational simulations. For instance, a study on imine-linked COFs employed PXRD pattern modeling to disambiguate between eclipsed (AA) and staggered (AB) stacking configurations by comparing experimentally obtained patterns with simulated profiles for each stacking mode [28,29]. Complementary to PXRD, TEM analysis enables direct visualization of stacking-related defects, as demonstrated in the high-resolution imaging of Tp-Azo COFs, which revealed stacking faults and dislocations critical for interpreting electronic properties [30]. Computational methods like density functional theory (DFT) further aid in distinguishing stacking arrangements by assessing their electronic band structures and energetics, as shown in studies on triazine-based COFs where band gaps were found to differ depending on the stacking sequence [31,32]. Thus, combining experimental and theoretical approaches is imperative for resolving stacking configurations and unlocking the full potential of these advanced materials.

### 2.2. Three-Dimensional Porphyrin COFs

Three-dimensional (3D) COFs are assembled from molecular building blocks that extend covalently in all three spatial directions to form open, periodic networks. Porphyrins and their metallated derivatives are highly conjugated π-macrocycles with distinctive photophysical and redox properties, and they serve as important molecular units in materials chemistry [26]. Embedding porphyrins into 3D COFs renders the porphyrinic sites addressable throughout the framework, endowing these materials with promise for gas storage and catalysis.

**Figure 4:**
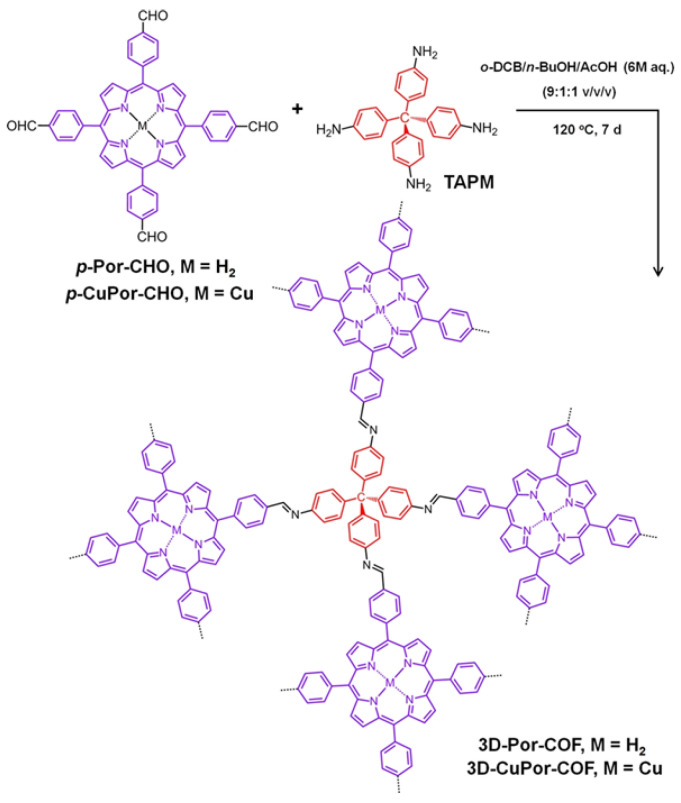
Representation of the Synthesis of 3D-Por-COF and 3D-CuPor-COF. Reproduced from [26], with permission from American Chemical Society, 2017.

Wang and co-workers [26] reported a targeted [4 + 4] imine-condensation strategy that couples a tetrahedral (3D-Td) node with a square (2D-C4) linker to afford two porphyrin-based 3D COFs: 3D-Por-COF and 3D-CuPor-COF (Figure 4). The crystalline nature of 3D COFs was confirmed by PXRD analysis. Both frameworks are microporous, exhibit high specific surface areas, and adopt a doubly interpenetrated topology indexed in the orthorhombic space group Pmc2_1_.

Zhang’s team [33] further employed an eight-connected organic linker with a four-connected porphyrin monomer in an [8 + 4] design to construct two 3D COFs featuring an scu topology, denoted NKCOFs-25-X (X = H or Ni). This approach broadens the toolkit of high-connectivity building blocks for 3D COFs and enriches the topological diversity accessible in porphyrin-based frameworks.

**Figure 5:**
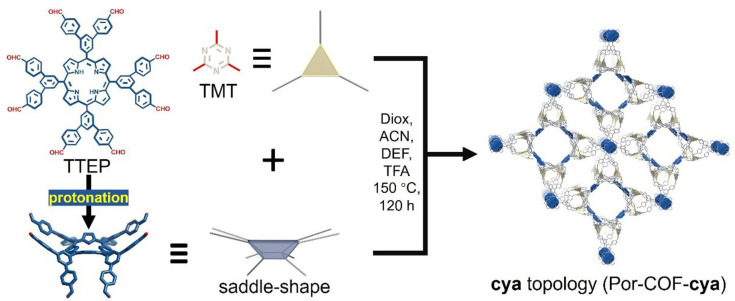
Synthesis of a porphyrin-based 3D COF of the cya topology between TTEP and TMT through Aldol condensation. Reproduced from [22], with permission from John Wiley and Sons, 2025.

Leveraging topology as a design handle, Jiang et al. [22] pre-encode a (3,8)-connected cya net into a vinylene-linked porphyrinic 3D-COF by aldol-coupling an eight-connected porphyrin node (TTEP) with a trigonal triazine linker (TMT), thereby realizing an unprecedented cya topology in molecule-based frameworks (Figure 5). PXRD with Pawley refinement and modeling identify a non-interpenetrated cubic lattice (space group PM3; a = b = c ≈ 40.996 Å), while acid-induced saddle (B2u) distortion of the porphyrin under synthesis conditions is shown to bias assembly toward the cya net—an instructive example of geometry-programmed topology. The framework is permanently porous (BET ≈ 1082 m^2^ g^−1^) with two hierarchical pore families (~10 and ~20 Å) that can host electron-rich guests such as tetrathiafulvalene (TTF), creating a donor–acceptor CTF@COF interface within the cya channels. Functionally, the topology-programmed host supports efficient charge separation/transport and high visible-light H_2_O_2_ production in water, which is further boosted upon TTF encapsulation. Collectively, Por-COF-cya showcases how predefining node connectivity and leveraging controllable macrocycle distortion can unlock new 3D-COF nets with programmable host–guest photoredox function.

**Figure 6:**
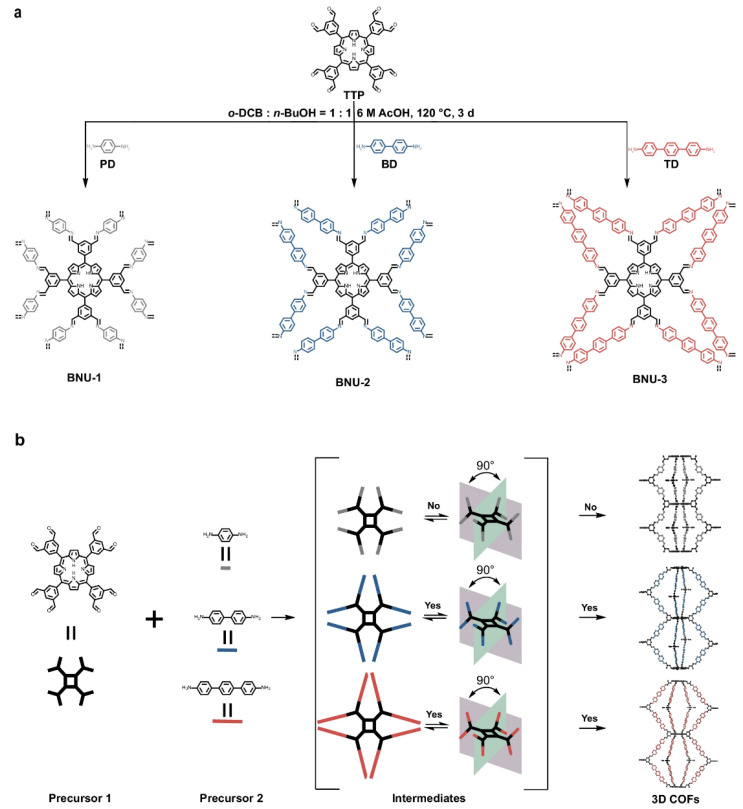
Schematic illustration of the synthetic strategy for 3D COFs. (**a**) Schematic representation of the synthesis of 3D COFs featuring porphyrin knots connected by linear amino π-conjugated linkers via C=N bonds. TTP, 5,5′,5″,5‴-(porphyrin-5,10,15,20-tetrayl)tetraisophthalaldehyde. PD, *p*-phenylenediamine. BD, benzidine. TD, 4,4″-diamino-*p*-terphenyl. (**b**) Mechanistic diagram illustrating the role of steric effects of intermediates in regulating the formation of 3D COFs. Black rods indicate porphyrin precursors, while gray, blue, and red rods represent linkers of varying lengths. Reproduced from [34], with permission from Springer Nature, 2025.

Mao et al. [34] propose an “intermediate-steric control” route to 3D-COFs: a multi-node porphyrin aldehyde (TTP; eight rotatable knots) is condensed with linear diamines so that the growing oligomeric intermediates rather than the precursors, adopt steric-hindrance–confined 3D conformations that guide crystallization into robust 3D frameworks (Figure 6). This concept departs from the prevailing need for intrinsically 3D precursors and enables self-adaptive growth toward diverse 3D nets. Demonstration with three linkers (PD/BD/TD) yields BNU-1/2/3; only the higher-hindrance pairs (TTP–BD/TD) give crystalline 3D pcb networks (2-fold interpenetrated) by PXRD/Rietveld refinement, whereas the low-hindrance TTP–PD route stalls into an amorphous solid—an outcome that tracks the designed topology diagram. Mechanistically, DFT-optimized models, flexible PES scans, and MD simulations show that increasing linker bulk drives meso-C–C rotation to a propeller-like porphyrin geometry (≈69° dihedrals for TTP-8BD/8TD vs. 65° for TTP-8PD), reduces conformational freedom, and replaces terminal H-bonding with π–π anchoring—conditions that favor ordered 3D extension at the eight nodes. Correlative microscopy/porosity data (HRTEM channel widths matching models; type-I isotherms with narrow pore distributions) further verify well-defined, interpenetrated 3D lattices. Collectively, intermediate-steric engineering introduces a generalizable control knob for 3D-COF synthesis, complementary to node-connectivity design, by leveraging the geometry and interactions of growth intermediates to program topology and crystallinity.

## 3. Synthesis of Porphyrin COFs

The synthesis of covalent organic frameworks (COFs) typically relies on the principles of dynamic covalent chemistry, in which reversible bond formation promotes crystallinity [24]. COF topology and porosity can be engineered by tuning the geometry of the monomers and their connection patterns. Owing to their planar structures and multifunctionality, porphyrinic linkers play a pivotal role in constructing π-conjugated, functional COFs [35]. With intrinsic C4 symmetry, porphyrins are topologically distinctive; most porphyrin-based COFs form two-dimensional sql networks via [4 + 2] condensations between C4-symmetric porphyrin nodes and linear linkers [36,37,38,39].

**Figure 7:**
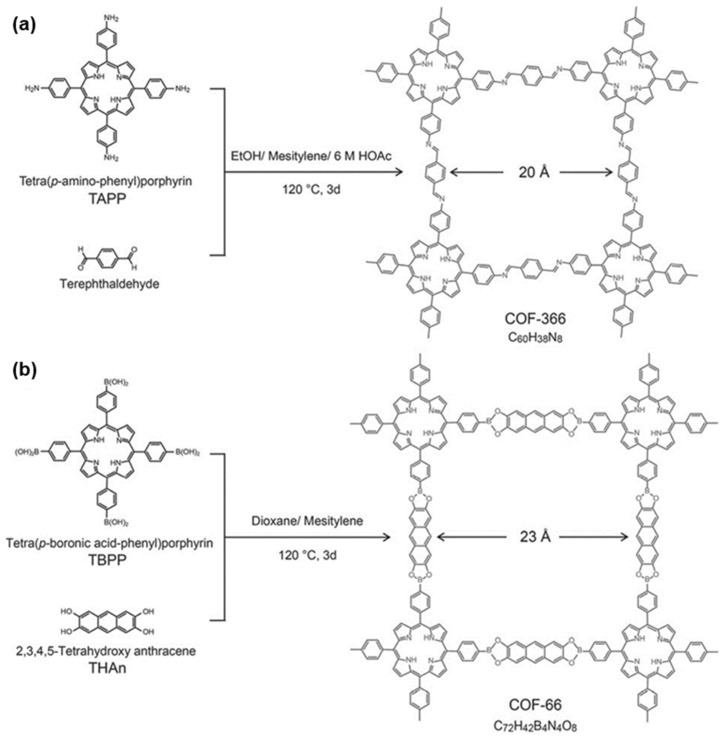
Condensation reactions between TAPP and terephthaldehyde, TBPP, and THAn. Produce Extended (**a**) COF-366 and (**b**) COF-66. Reproduced from [39], with permission from American Chemical Society, 2011.

For instance, the Yaghi group [39] synthesized two porphyrin COFs—COF-366 and COF-66—by solvothermal condensation (Figure 7). COF-366 is built through a [4 + 2] reaction between a square linker, 5,10,15,20-tetrakis(4-aminophenyl)porphyrin (TAPP), serving as the vertex, and a linear linker, terephthalaldehyde (TPA), serving as the edge, to furnish a 2D sql lattice. The crystalline nature of the synthesized COFs were confirmed by PXRD analysis. Likewise, 5,10,15,20-tetrakis(4-formylphenyl)porphyrin (TFPP) can act as the vertex to assemble sql frameworks with *p*-phenylenediamine as the linker [19]. Within the framework of reticular chemistry, the “edges” of COF-366 can be elongated by replacing TPA with extended linkers such as 2,3,4,5-tetrahydroxyanthracene (THAn) or 4,4′-biphenyldicarboxaldehyde (BPDA), thus producing the sql networks COF-66 and COF-367, respectively [39,40]. Christopher et al. [40] further prepared imine-linked porphyrin COFs by condensing 2,3-dihydroxyterephthalaldehyde with 5,10,15,20-tetrakis(4-aminophenyl) metalloporphyrins, combining C4-symmetric porphyrins with C2-symmetric linkers to yield quadrilateral COFs with sql topology.

**Figure 8:**
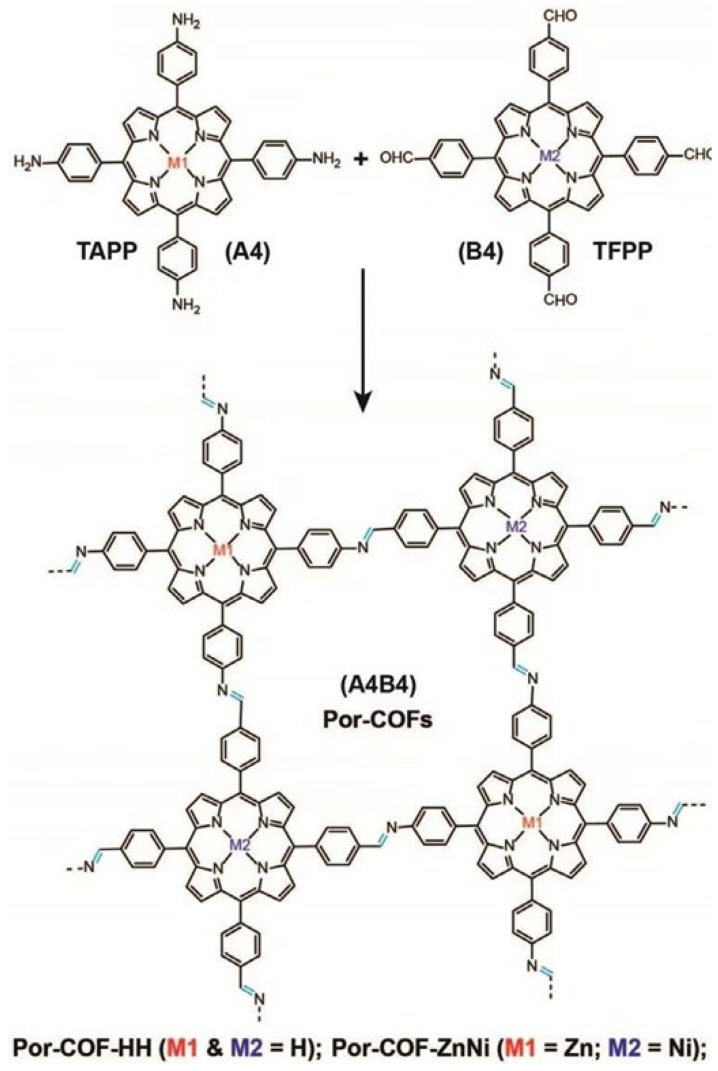
Representations of the synthesis of Por-COFs(M1&M2=H): reacted in the presence of aqueous acetic acid (6 M) using *o*-dichlorobenzene and *n*-butanol (1:1 *v*/*v*) as the solvent combination at 120 °C for 3 days. Reproduced from [41], with permission from John Wiley and Sons, 2019.

Porphyrin COFs can also be constructed via [4 + 4] strategies. Biswal and co-workers [41] reported an imine-linked porphyrin COF with an extended π-conjugated backbone, Por-COF-HH (Figure 8), formed by the Schiff-base condensation of TFPP with TAPP. Lan’s group [42] employed a hydrothermal [4 + 4] imine condensation between nickel phthalocyanine octacarboxylic acid (NiPc) and 5,10,15,20-tetrakis(4-aminophenyl)porphyrin (2HPor) to afford NiPc-2HPor COF. These porphyrinic COFs (Por-COFs) exhibit high crystallinity and permanent porosity while preserving the characteristic sql lattice. The crystal structures of these COFs were characterized by PXRD measurement and simulations.

**Figure 9:**
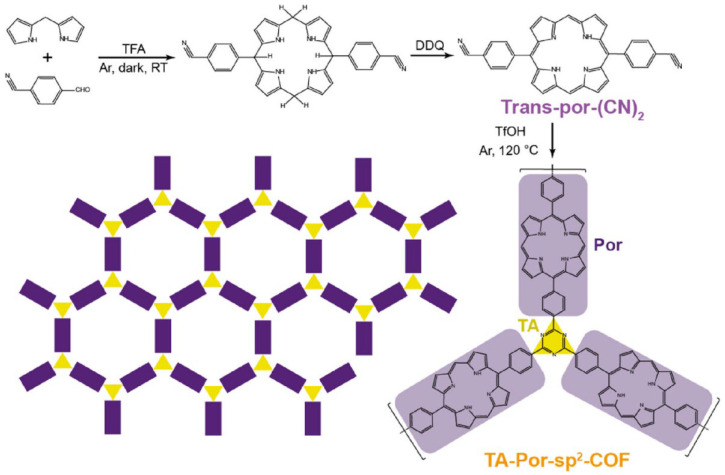
Synthetic route and structure of the triazine–porphyrin-based hyper-conjugated COF. Reproduced from [12], with permission from American Chemical Society, 2022.

Beyond imine bonds, boronate ester and triazine linkages are also frequently employed to construct porphyrinic COFs [43,44,45]. The reversible condensation of boronic acids with catecholates enables the formation of five-membered boronate esters and six-membered boroxine rings, facilitating crystallization [13]. For example, Dong et al. [46] synthesized a 2D porphyrin COF with sql topology via a [4 + 2] condensation between 5,10,15,20-tetrakis [4-(dihydroxyboryl)phenyl]porphyrin (TBP) and 1,2,4,5-tetrahydroxybenzene (THB), linked by boronate esters. Yu et al. designed a triazine-linked, sp^2^-carbon–conjugated porphyrin COF using a novel trans-A2B2 porphyrin monomer [Trans-por(CN)_2_]; cyano self-polymerization afforded the crystalline, periodic framework TA-Por-sp^2^-COF (Figure 9) [12]. PXRD analysis was employed to disclose the crystallinity of these COFs. In general, boronate ester linkages are typically more sensitive to hydrolysis, while imine and especially triazine linkages generally provide higher chemical and thermal robustness owing to their strong C=N bonding.

The repertoire of covalent linkages in COF synthesis has continued to expand. Li and co-workers obtained an amide-linked 2D Por-PD-COF from tetrakis(4-carboxyphenyl)porphyrin (TCPP) and *p*-phenylenediamine (PD) via a condensation route; the material combines straightforward preparation with efficient adsorption, high selectivity, and recyclability, suggesting utility in targeted organic separations and photocatalysis [47]. Electrochemical deposition provides a convenient pathway to porphyrinic COFs: Tavakoli et al. [13] electrodeposited 5,10,15,20-tetrakis(4-aminophenyl)porphyrin-based COFs, forging phenazine linkages under controlled conditions and demonstrating a bottom-up, rapid strategy to grow COF dendrites. In addition, Yamamoto coupling (dehalogenative C–C coupling) has proven effective for fully aryl-linked COFs, enabling the synthesis of porphyrin frameworks from tetrakis(4-bromobiphenyl) porphyrin precursors [47,48]. Collectively, C–C coupling, aldol-type condensations, and Schiff-base reactions now make it possible to design and access highly crystalline porphyrin COFs with diverse topologies.

**Figure 10:**
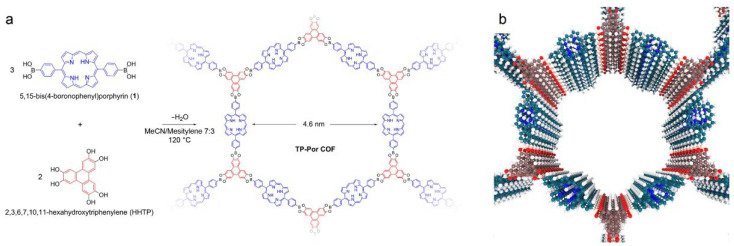
(**a**) Co-condensation of bis(boronophenyl)porphyrin 1 and HHTP leading to the formation of the layered TP-Por COF. The COF features hexagonal pores with a large diameter of 4.6 nm. (**b**) Illustration of the TP-Por COF highlighting the alternating columns of triphenylene (red) and porphyrin (blue) subunits. Reproduced from [37], with permission from American Chemical Society, 2014.

While most porphyrinic COFs adopt sql lattices, judicious control over building-block geometry unlocks a wide array of network topologies [35]. Calik et al. [37] used a solvothermal [2 + 3] condensation between a linear linker, 5,15-bis(4-boronophenyl)porphyrin (BBP), and a triangular node, 2,3,6,7,10,11-hexahydroxytriphenylene (HHTP), to yield a 2D TP-Por COF with hcb (hexagonal) topology (Figure 10), featuring porous hexagonal channels with triphenylene vertices, porphyrinic edges, and periodic structure confirmed by PXRD. Wang and colleagues [15] employed a [4 + 4] imine condensation of a square linker (TFPP) with a tetrahedral node (tetra(4-aminophenyl)methane, TAPM) to produce the first 3D porphyrin COF with pts topology (3D-Por-COF), whose microporosity and doubly interpenetrated framework suggest new design principles for 3D porphyrinic COFs. More recently, Zhang et al. [33] combined an eight-connected organic linker with a four-connected porphyrin unit in an [8 + 4] strategy to construct two 3D COFs with scu topology, further enriching both the building-block toolbox and the topological diversity of porphyrin-based frameworks.

Obtaining crystalline COFs is a labor-intensive process that requires meticulous control over reaction conditions due to inherent challenges related to their crystallization. One of the primary issues stems from the reversible nature of covalent bond formation, such as imine, boronate ester, or ketoenamine linkages, which is critical for error correction during self-assembly but can also lead to slow and incomplete reactions. Inconsistent operation of these reversible linkages can hinder the formation of long-range order necessary for high crystallinity. For instance, imine-linked COFs often require precise tuning of reaction conditions, as seen in studies that utilized modulators like acetic acid to balance reaction kinetics and enhance reversibility [49]. The choice of solvent also plays a key role; high-boiling solvent mixtures such as mesitylene/dioxane have been shown to improve crystallization by enabling extended periods of bond exchange and self-healing during nucleation and growth [49]. Furthermore, temperature sensitivity is a complicating factor, with deviations often accelerating side reactions or leading to amorphous byproducts, mandating strict thermal control. Modulators, including acidic or basic additives, are essential for fine-tuning pH levels and reaction rates, as demonstrated in the synthesis of boronate ester-based COFs using trifluoroacetic acid to stabilize crystallization [50]. Overall, the optimization of these interdependent parameters through iterative experimentation is often necessary to achieve highly crystalline COFs suitable for advanced applications.

## 4. Applications

### 4.1. CO_2_ Capture and Storage

Large CO_2_ emissions from fossil-fuel combustion are a principal driver of global warming, underscoring the need for efficient capture and storage technologies [51]. Although ordered, open channels in 2D COFs are favorable for CO_2_ adsorption, their tightly stacked layers and modest porosity can limit uptake [52,53,54,55]. Materials whose pore apertures match the kinetic diameter of CO_2_ (≈3.3 Å) typically exhibit superior capacities [56]. The rigid porphyrin macrocycle endows COFs with relatively large, well-defined pores that are amenable to post-synthetic wall functionalization, offering an excellent 2D platform for CO_2_ capture.

**Figure 11:**
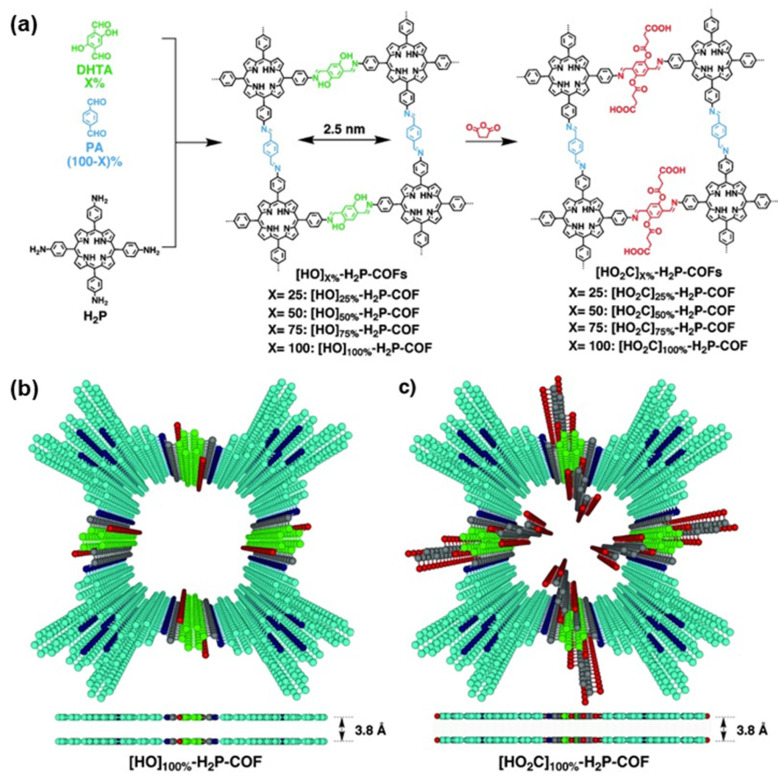
(**a**) Synthesis of [HO]X%-H_2_P-COFs: reacted in a mixed solvent of *o*-dichlorobenzene/*n*-butanol/6 M acetic acid (5/5/1 by vol.) at 120 °C for 3 days. The subsequent functionalization of channel walls with succinic anhydride in anhydrous acetone at 60 °C for 2 days, leading to [HO_2_C]X%-H2P-COFs. (**b**) [HO]_100%_-H_2_P-COF and (**c**) [HO_2_C]_100%_-H_2_P-COF. Reproduced from [52], with permission from John Wiley and Sons, 2015.

In 2015, Huang and co-workers [52] prepared functionalized COFs by co-condensing 2,5-dihydroxyterephthalaldehyde (DHTA), phthalaldehyde (PA), and a porphyrin vertex (H_2_P), affording [HO]X%-H_2_P-COFs (X = 25, 50, 75, 100) (Figure 11). While these frameworks adsorbed CO_2_ to a limited extent, subsequent treatment with succinic anhydride quantitatively introduced carboxylic acids onto the channel walls to give [HO_2_C]X%-H_2_P-COFs (X = 25, 50, 75, 100) (Figure 10). This modification preserved the layered, open porosity yet markedly enhanced uptake; [HO_2_C]100%-H_2_P-COF reached 174 mg·g^−1^ at 1 bar. This strategy provides a route to convert conventional 2D materials into efficient CO_2_-capture scaffolds and broadens the application space of 2D COFs in high-performance gas storage and separation.

**Figure 12:**
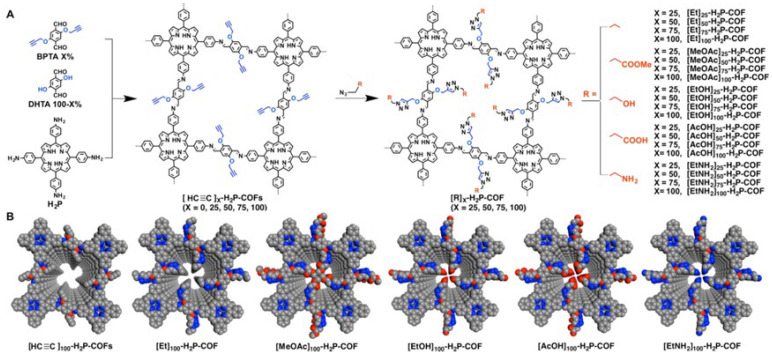
(**A**) Schematic of Pore Surface Engineering of Imine-Linked COFs with Various Functional Groups via Click Reactions: reacted in a mixed solvent of *o*-dichlorobenzene/*n*-butanol/3 M acetic acid (5/5/1 by vol.) at 120 °C for 3 days, then reacted in a mixed solvent of toluene/*n*-butanol (4/1 by vol.) in the presence of CuI and DIPEA at room temperature for 24 h; (**B**) Pore Structures of COFs with Different Functional Groups (Gray, C; Blue, N; Red, O) Carbon dioxide adsorption capacity of the COFs at 273 (red) and 298 K (blue) and 1 bar. Reproduced from [56], with permission from American Chemical Society, 2015.

Extending this concept, the same group [56] started from a mesoporous, imine-linked porphyrin COF with low CO_2_ uptake and built a three-component system comprising 5,10,15,20-tetrakis(4-ethynylphenyl)porphyrin (H_2_P), 2,5-bis(2-propoxy)terephthalaldehyde (BPTA), and DHTA to generate [HC≡C]X%-H_2_P-COFs (X = 0, 25, 50, 75, 100) (Figure 12). Quantitative azide–alkyne “click” reactions then immobilized diverse pendant groups on the pore walls, yielding a library of 20 COFs with PXRD determined crystallinity (Figure 12A) whose channels were tailored by integrating different functionalities (Figure 12B). CO_2_ uptake correlated with the nature of the grafted groups: relative to nonpolar ethyl pendants, polar moieties significantly increased adsorption. In particular, carbonyl and hydroxyl groups enhanced uptake via dipolar/electrostatic interactions, while aminoethyl pendants formed acid–base pairs and delivered especially high capacities. For example, [EtNH_2_]50-H_2_P-COF adsorbed 157 and 82 mg·g^−1^ CO_2_ at 273 and 298 K, respectively. These studies highlight porphyrin COFs as versatile platforms for pore-surface functionalization to boost CO_2_ capture, with clear implications for environmental remediation.

In the context of CO_2_ capture, porphyrin-based COFs generally exhibit moderate-to-high adsorption capacities that scale with surface area, pore size, and wall polarity, comparable to those of other functional COFs with similar textural properties [52,56,57]. Their distinctive advantage emerges when CO_2_ capture is coupled to photocatalytic or electrocatalytic reduction: porphyrin macrocycles act as light-harvesting antennas, redox-active scaffolds, and, upon metallation, well-defined M–N_4_ catalytic sites embedded in an ordered porous framework. As illustrated by representative systems in this review, porphyrin-based COFs deliver CO_2_ reduction activities, product selectivities, and operational stabilities that are competitive with, and in several cases superior to, those of non-porphyrinic COFs and molecular porphyrin benchmarks under comparable conditions.

### 4.2. Catalytic Reduction Reaction of CO_2_ (CO_2_RR)

#### 4.2.1. Photocatalysis

Covalent organic frameworks (COFs) offer an attractive platform for CO_2_ reduction owing to their intrinsic porosity, large specific surface areas, platform for post-functionalization and architecturally tunable backbones [23,58,59,60,61,62,63]. Their abundant pores and high densities of heteroatoms often confer stronger CO_2_ affinity and selectivity than conventional semiconductors [64,65,66,67,68,69,70]. Porphyrin derivatives have long served as efficient, selective CO_2_RR catalysts [71,72]; embedding porphyrin units into COF channels combines these catalytic advantages with ordered mass/charge transport in a single material [23].

Lan et al. [65] designed a series of 2D porphyrin COFs via solvothermal Schiff-base condensation between metallated 5,10,15,20-tetrakis(4-aminophenyl)porphyrin (TAPP) and a tetrathiafulvalene (TTF)-based linker, 2,3,6,7-tetrakis(4-methylphenyl)TTF. Electron-deficient metalloporphyrins derived from TAPP provide strong visible-light harvesting and inherent CO_2_-reduction activity [73], whereas the electron-rich TTF unit is an excellent, fast electron donor [74,75,76]. Effective covalent coupling between TAPP and TTF in the COF promotes visible-light-driven electron transfer from TTF to TAPP, localizing photogenerated electrons on the porphyrin for CO_2_ reduction and holes on the TTF for H_2_O oxidation. This work constitutes the first report of a rationally designed porphyrin COF that uses H_2_O as the electron donor for selective photoreduction of CO_2_.

**Figure 13:**
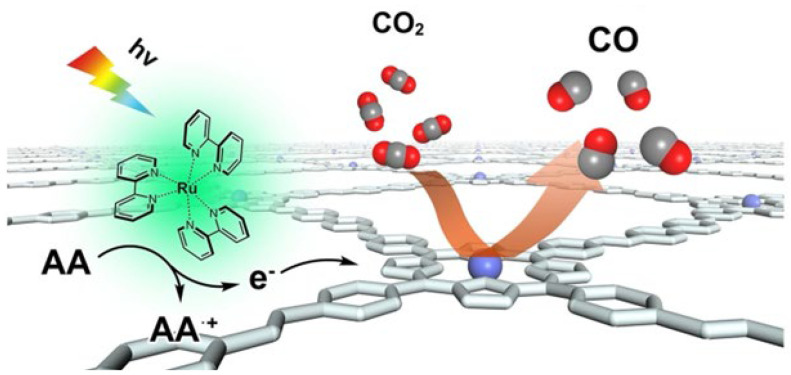
Proposed mechanism for the photocatalytic conversion of CO_2_ into CO over COF-367-Co NSs under visible-light irradiation with [Ru(bpy)_3_]^2+^ as the photosensitizer and AA as the electron donor. Reproduced from [67], with permission from American Chemical Society, 2019.

Subsequently, the same team [67] prepared ultrathin porphyrin-COF nanosheets (NSs), COF-367-Co, via imine-exchange. The crystalline nature of the COF Ns material was characterized by PXRD. Because both free-base and metalloporphyrin units in the COF NSs serve as catalytically active sites under heterogeneous photocatalytic conditions, the Co-porphyrin COF nanosheets were employed as catalysts for CO_2_-to-CO conversion using [Ru(bpy)_3_]^2+^ as a photosensitizer and ascorbic acid as a sacrificial agent (Figure 13). Under visible light, the ultrathin 2D COF-367-Co NSs expose a large number of accessible sites, thereby enhancing the photocatalytic reduction of CO_2_ to CO.

**Figure 14:**
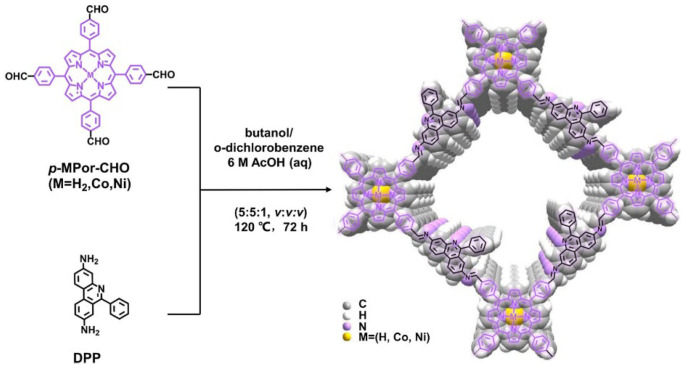
Schematic Illustration Showing the Structure of MPor-DPP-COF. Reproduced from [77], with permission from American Chemical Society, 2022.

Wang et al. [77] condensed a porphyrin aldehyde (*p*-MPor-CHO) with a 3,8-diamino-6-phenyl DPP linker to obtain a family of 2D porphyrinic COFs (MPor-DPP-COFs) (Figure 14). The crystalline nature of MPor-DPP-COFs was clearly revealed by PXRD analysis results. The bulky peripheral phenyl substituents around the DPP unit effectively suppress interlayer π–π stacking, enlarging the gallery spacing and increasing the accessible surface area. This structural modulation affords more exposed active sites and facilitates mass/charge transport, enabling the Co-incorporated framework (Co-DPP-COF) to exhibit promising photocatalytic activity for CO_2_RR. Tuning the interlayer spacing thus emerges as a viable handle to boost CO_2_-to-CO conversion efficiency in porphyrin COFs.

**Figure 15:**
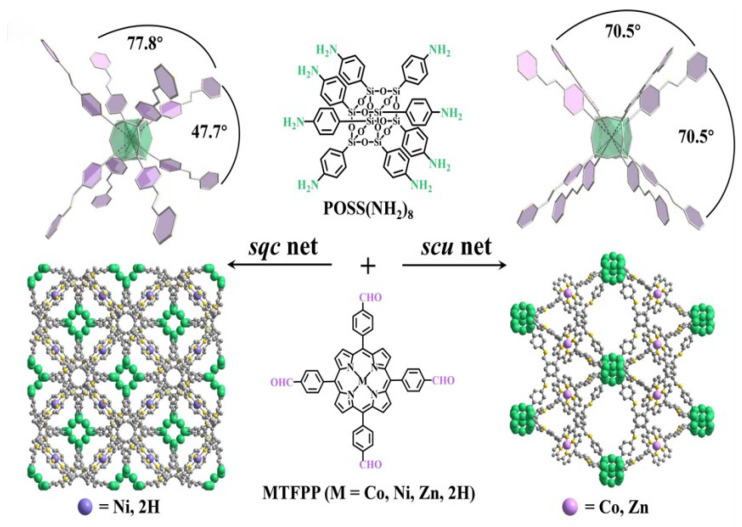
Synthesis of 3D COFs with the sqc and scu topology. Reproduced from [78], with permission from John Wiley and Sons, 2024.

Jiang et al. [78] created a cubic octa-aminophenyl silsesquioxane node, POSS(NH_2_)_8_, with 5,10,15,20-tetrakis(4-methylphenyl)porphyrins (MTFPP; M = Co, Zn, Ni, H) to access two topological families: POSS-MTFPP-COFs-scu (M = Co, Zn) and POSS-MTFPP-COFs-sqc (M = Ni, H). Incorporation of porphyrinic sites endowed these frameworks with strong activity for the visible-light (λ > 420 nm) photocatalytic reduction of CO_2_ to CO (Figure 15). PXRD results revealed the crystalline structures of the COFs. Among them, POSS-NiTFPP-COF-sqc delivered the best performance, achieving a CO formation rate of 9680 mmol h^−1^ with 97% selectivity. The inferior activity of POSS-H_2_TFPP-COF-sqc is attributed to the absence of metal adsorption sites. In contrast, Ni(II) centers in the sqc lattice show high CO_2_ affinity and, together with the smaller pore apertures of the sqc framework, enrich CO_2_ in the vicinity of Ni sites, enhancing turnover. Although the scu networks possess larger pores that promote bulk CO_2_ uptake, the lower intrinsic CO_2_ affinity of Co(II) and Zn(II) relative to Ni(II) ultimately limits the selectivity of POSS-MTFPP-COFs-scu compared with POSS-NiTFPP-COF-sqc.

**Figure 16:**
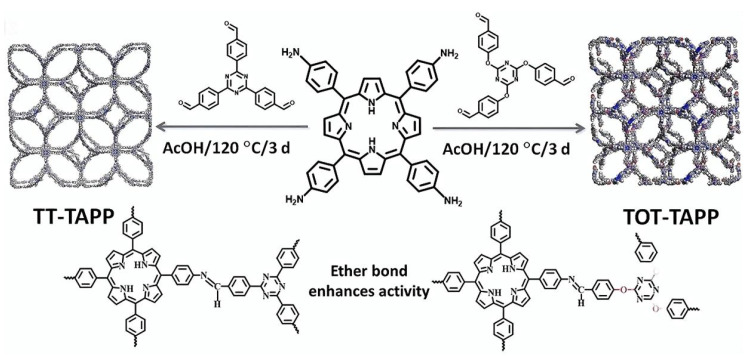
Schematic representation of the synthesis of TOT-TAPP and TT-TAPP COFs. Reproduced from [79], with permission from John Wiley and Sons, 2025.

Targeting the long-standing challenge of steering photogenerated electrons to the CO_2_ adsorption locus, Liu et al. [79] embed ether linkages into porphyrin–triazine COFs (TOT-TAPP, BOD-TAPP, QOB-TAPP) to regulate charge-transfer pathways and co-localize the electron-enriched site with the preferred CO_2_ binding site (the imine C=N) within the same channel (Figure 16). The crystallinity of the prepared COFs was assessed by PXRD. In situ and spectroscopic evidence converge on this “electron-funneling” effect: ether insertion accelerates carrier separation and drives a pronounced electron build-up at C=N (N 1s/C=N binding-energy decrease by ~0.4 eV under illumination), while fs-TAS and PL/TRPL show longer carrier lifetimes (τ_e_ ≈ 10.96 ps vs. 7.33 ps; 3.11 ns vs. 1.04 ns for TOT-TAPP vs. TT-TAPP), consistent with enhanced charge transport corroborated by smaller EIS semicircles and stronger transient photocurrents. Performance tracks mechanism: under full-spectrum Xe-lamp irradiation, TOT-TAPP doubles the CO production rate relative to the ether-free control (34.8 vs. 18.0 μmol g^−1^ h^−1^), and the BOD/QOB analogues likewise outperform their non-ether counterparts, all with high CO selectivity and robust cycling stability. In situ DRIFTS captures carbonate/bicarbonate adlayers and the *COOH intermediate, and DFT assigns a reduced barrier for the *CO-forming rate-determining step on TOT-TAPP. Notably, metalating the porphyrin macrocycle degrades activity by misaligning the electron-accumulation zone with the CO_2_ adsorption site, underscoring that spatial alignment of electron-rich and adsorption sites.

Advancing structure–function control in Por-COFs, Jiang et al. [21] engineered a family of 2D cobalt-porphyrin frameworks whose interlayer planarity is deliberately disrupted by twisted linkers to expose buried sites while preserving long-range order. In the N–N–bicarbazole variant (NN-Por-COF), steric clash across a rotatable N–N bond drives a markedly undulated stacking (carbazole–carbazole dihedral ≈ 72.9°, enlarged interlayer spacing), as verified by PXRD/Pawley refinement, which in turn increases Co-site accessibility and facilitates mass transport. Photophysically, NN-Por-COF shows stronger photosensitizer quenching and shorter TRPL lifetimes than its less-undulated analogues (CC-Por-COF, C/C-Por-COF), indicating more efficient charge separation/transfer. Catalytically, under visible light with [Ru(bpy)_3_]^2+^/TIPA in MeCN/H_2_O, NN-Por-COF achieves CO evolution rates of 22.38 mmol g^−1^ h^−1^ (pure CO_2_) and 3.02 mmol g^−1^ h^−1^ (10% CO_2_), outperforming the flatter controls and ranking among the best Por-photocatalysts reported under comparable conditions. Control experiments isolate the structural and site effects: an amorphous NN-Por polymer is markedly slower, and the metal-free NN-H_2_Por-COF is essentially inactive; isotope labeling confirms CO originates from CO_2_, while durability, AQE (0.61% at 420 nm), and post-reaction characterization support robustness. Together, these data delineate a clear design rule—introducing undulation into 2D Por-COFs simultaneously amplifies active-site exposure and optimizes Co-center electronics, translating to faster CO_2_-to-CO photoreduction across concentrated and dilute feeds.

Wang et al. [80] encapsulate fullerene C70 into a tetrathiafulvalene–Co-porphyrin imine COF (TTF–CoTPP), yielding a donor–acceptor C70@COF that broadens the absorption edge and redirects the LUMO onto C70, thereby establishing an internal electron-accepting pathway. Femtosecond transient absorption resolves ultrafast (≈0.3 ps) formation of a charge-separated state (TTF–CoTPP^•+^–C70^•−^), followed by a hole-shift to TTF that produces TTF^•+^–CoTPP–C70^•−^ with a lifetime > 5 ns; this multistep sequence is consistent with stronger photocurrent/SPV–TPV responses and enhanced PL quenching. Mechanistically, in situ DRIFTS captures *COOH and *CO surface intermediates, while DFT shows that C70 lowers the barrier for the rate-determining *CO_2_→*COOH step (1.30→1.16 eV), rationalizing the accelerated CO_2_-to-CO kinetics. Under Ru(bpy)_3_^2+^/TEOA in MeCN/H_2_O, C70@COF reaches a CO formation rate of 4.96 × 10^3^ μmol g^−1^ h^−1^ (1.95 times pristine COF), ranks among the better COF photocatalysts under comparable conditions, maintains crystallinity/morphology over cycling, and delivers an AQY of 0.41% at 450 nm. Collectively, pore-confined fullerene engineering furnishes a clear structure–mechanism–performance link—multistep charge transfer that sustains long-lived separation—providing a generalizable blueprint for boosting CO_2_ photoreduction in Por-COF platforms.

Extending the “site–transport co-programming” paradigm to 3D Por-COFs, Yuan et al. [81] design an 8-connected porphyrinic node (TTPP) and couple it with three linear dialdehydes of increasing conjugation/electron richness (Bpd → Tpd → benzimidazole-containing BFBie) to build interpenetrated bcu networks whose linker “electron-cloud density” can be dialed a priori. Structural and spectroscopic analyses confirm crystalline 2-fold-interpenetrated frameworks with imine formation (FT-IR/^13^C NMR) and resolved PXRD reflections indexed by simulation, establishing a robust platform for electronic tuning. Mechanistically, increasing π-delocalization from Bpd/Tpd to BFBie elevates local electron density along the conjugated paths, while cobaltation of the porphyrin centers and post-synthetic imine protonation further reshape the charge landscape—both strategies measurably promote charge separation and carrier migration within the lattice. Photoelectrochemical diagnostics align with this picture: among the series, COF-3-Co (BFBie linker) exhibits the smallest optical gap, the most negative LUMO vs. CO_2_/CO, the lowest EIS charge-transfer resistance, and the strongest transient photocurrent, with Mott–Schottky plots confirming n-type character; protonation further boosts photocurrent and lowers interfacial resistance. Performance tracks the electronics: under visible light, COF-3-Co delivers the highest CO yield (23.17 mmol g^−1^) with ~94% selectivity, and its protonated analogue HCOF-3-Co climbs to 30.44 mmol g^−1^ (with a modest selectivity drop to ~87% due to enhanced HER competitiveness). The reaction pathway is corroborated by isotope tracing (^13^CO at *m*/*z* = 29) and in situ ATR-FTIR that captures *COOH/*CO and multiple carbonate adlayers, consistent with CO_2_-to-CO conversion via the *COOH route. Collectively, this work establishes “electron-cloud-density modulation” of 3D Por-COFs—via linker electronics, metalation, and protonation—as an effective knob to synchronize band energetics and interfacial transport, thereby accelerating CO_2_ photoreduction on porphyrinic frameworks.

#### 4.2.2. Electrocatalysis

While the studies above center on photocatalytic CO_2_ reduction in porphyrin COFs, electrocatalytic CO_2_ reduction is an equally promising strategy—especially when paired with anodic organic oxidation to co-produce value-added chemicals and improve overall energy efficiency. In 2015, Christopher and co-workers [40] tethered cobalt porphyrins to a COF via organic side chains to create tunable catalytic sites. By modulating the electronic environment of the Co centers within the framework, they obtained a new electrocatalyst with enhanced activity, stability, and selectivity, achieving a Faradaic efficiency of ~90% and a turnover number up to 29,000 for the selective reduction of CO_2_ to CO in aqueous electrolyte.

Building on this concept, Zhang et al. [82] synthesized, by an ionothermal route, a porphyrin-based covalent triazine framework (NiPor-CTF) featuring abundant, atomically dispersed NiN_4_ centers. The fully conjugated backbone enriched with triazine nodes facilitates charge transport and ion adsorption. As an electrocatalyst for CO_2_RR, NiPor-CTF delivered outstanding selectivity over approximately −0.6 to −0.9 V and maintained excellent long-term stability. This superior performance arises from the synergistic effect of a high density of exposed NiN_4_ active sites and the strong CO_2_-capturing capability of the triazine motifs. Overall, these studies highlight a modular design paradigm in which the molecular scaffold surrounding well-defined M–N_4_ sites can be precisely engineered to boost CO_2_RR selectivity and efficiency.

Zhuang and co-workers [83] employed an ionothermal strategy in molten ZnCl_2_ to polycondense 5,10,15,20-tetrakis(4-cyanophenyl)porphyrin (Por) and its nickel complex (NiPor), affording a NiPor-based covalent triazine framework (NiPor-CTF) and, for comparison, a Por-CTF. Further pyrolysis of NiPor-CTF at 900 °C under N_2_ yielded Ni/N-rich porous carbon (Ni/N-PC). These materials feature abundant NiN_4_ active sites, fully conjugated backbones, and triazine-rich nodes that facilitate charge transport and ion adsorption. This straightforward, building-block design is readily extendable to multifunctional porous organic electrocatalysts. Electrochemically, NiPor-CTF delivered a current density of 52.9 mA cm^−2^ at −0.9 V, surpassing Ni/N-PC (37.1 mA cm^−2^) and Por-CTF (6.2 mA cm^−2^), indicating higher intrinsic activity. Its Faradaic efficiency for CO exceeded 90% across −0.6 to −0.9 V, peaking at 97% at −0.9 V; Ni/N-PC showed intermediate selectivity, while Por-CTF was the lowest. The partial current densities for CO on NiPor-CTF and Ni/N-PC were consistently larger than on Por-CTF over the tested potentials. Overall, the selectivity trend NiPor-CTF > Ni/N-PC ≫ Por-CTF underscores the pivotal role of NiN_4_ sites. The exceptional CO_2_-to-CO selectivity of NiPor-CTF highlights how coordinating well-defined active centers within an optimized molecular scaffold can markedly enhance CO_2_RR efficiency and selectivity, offering a modular blueprint for the precise design of multifunctional electrocatalysts.

Lan and co-workers [42] hydrothermally constructed a crystalline, π-stacked NiPc–2HPor COF and, via post-synthetic coordination, converted it to a bimetallic NiPc–NiPor COF. The resulting ordered, permanently porous networks feature highly π-conjugated backbones that facilitate charge transport and confer comparatively high electronic conductivity—attributes well suited to electrocatalysis. Under paired electrolysis (cathodic CO_2_ reduction coupled with anodic alcohol oxidation), the NiPc–NiPor COF—hosting two Ni sites in distinct chemical environments—delivered higher Faradaic efficiencies and larger partial current densities (j) for both the methanol oxidation reaction (MOR) and the ethanol oxidation reaction (EOR) than its mono-metalated analogue. In methanolic electrolyte, both NiPc and NiPor motifs enhance MOR currents; however, linear sweep voltammetry (LSV) of the isolated monomers indicates that NiPc is intrinsically more active than NiPor for EOR, underscoring the decisive role of the NiPc node. With increasing potential, currents recorded for NiPc–2HPor and NiPc–NiPor COFs in methanolic media exceed those in neat KOH, and NiPc–NiPor consistently outperforms NiPc–2HPor, implicating NiPc units as key promoters of MOR. Moreover, EOR measurements in Ar- and CO_2_-saturated electrolytes yield nearly identical current densities for the two COFs under CO_2_, further supporting that EOR activity arises predominantly from NiPc rather than NiPor. Collectively, among the materials surveyed, NiPc–NiPor COF exhibits the most favorable balance of MOR activity and selectivity, validating the strategy of integrating dual, functionally differentiated Ni sites into a conductive COF scaffold.

Wang et al. [84] synthesized a three-dimensional, interpenetrated tbo-type COF (3D Co/Ni-TPNB-COF) by linking Co(II)- and Ni(II)-porphyrin units through a tris(1,1′-biphenyl)-4,4″,4‴-amine (NB) node. The NB junction promotes through-framework charge transport and redistributes charge density between the porphyrinic sites, engendering a high density of redox-active centers. In neutral electrolyte the material delivers a high Faradaic efficiency (FE) for CO_2_-to-CO (~95%) together with an excellent turnover frequency (TOF = 4.10 s^−1^). Electrochemical diagnostics—LSV, CV, Tafel slopes, and EIS—consistently indicate advantages in activity, selectivity, charge-transfer kinetics, and durability, positioning this 3D architecture as an effective and economically attractive CO_2_-reduction platform.

Van Der Veen et al. [85] prepared a family of Ni/Zn-porphyrin COFs by solvothermal polycondensation of Ni-TAPP and Zn-TAPP with 2,5-dihydroxyterephthalic acid (DHTA), systematically varying the Ni:Zn ratio (Ni100/Zn0→Ni0/Zn100). A clear compositional synergy emerged in CO_2_RR: the equimolar Ni50/Zn50 COF—exhibiting the highest fractions of micro- and mesopores—afforded the greatest CH_4_ production rate and overall catalytic activity, whereas the Ni- and Zn-rich end members favored H_2_ and CO, respectively. Thus, tuning the Ni:Zn porphyrin ratio provides a practical handle for product-selectivity control and a general route to bifunctional (and potentially higher-order multifunctional) COF electrocatalysts.

Downsizing et al. [86] introduced a morphology-control strategy to tailor porphyrin-COF particle size. Cobalt porphyrin (Co(TAPP)) was first tritylated with triphenylmethyl bromide to give Co(ttpp); subsequent solvothermal reaction with terephthalaldehyde in nitrobenzene/1,4-dioxane (1:3) in the presence of benzoic acid and water enabled in situ detritylation and colloidal deprotection–polycondensation, furnishing COF-T. Relative to COFs from unprotected monomers (COF-A), COF-T formed smaller, less-aggregated particles while retaining crystallinity and porosity, which translated into superior CO_2_RR metrics in 0.5 M KHCO_3_(aq): a higher initial TOF and a higher, more stable FE(CO) of 86–95% (vs. 38–67% for COF-A), with comparable durability. The data indicate that reducing particle size primarily enhances interfacial contact with the conductive additive, thereby boosting CO production and TOF, and underscore particle-size control as a generalizable lever for performance tuning.

**Figure 17:**
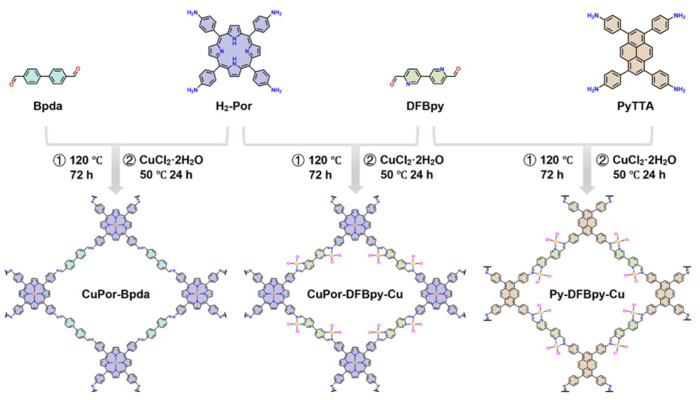
Syntheses and structures of CuPor-Bpda, CuPor-DFBpy-Cu, and Py-DFBpy-Cu. Reproduced from [87], with permission from John Wiley and Sons, 2025.

Fan et al. [87] architect a porphyrin–bipyridine COF that co-hosts two isolated Cu single-site chemistries with CuN_4_ on the porphyrin macrocycle and CuN_2_Cl_2_ on an imine–bipyridine pocket, yielding the dual-site catalyst CuPor-DFBpy-Cu (Figure 17). In contrast to the other metalloporphyrin COFs discussed here, this dual-site CuPor-DFBpy-Cu is obtained via a post-synthetic metalation step applied to a porphyrin–bipyridine framework, making the synthesis does not rely solely on pre-metallated porphyrin monomers. Crystallographic analysis (PXRD/Pawley) resolves an AA-stacked Cmmm lattice with a ≈ 40.9 Å, b ≈ 44.2 Å, c ≈ 3.51 Å, while HRTEM shows honeycomb channels; all three comparison COFs share similar diffraction signatures and high surface areas (S_BET_ ≈ 1121–1237 m^2^ g^−1^). Functionally, the dual-site framework delivers methane-selective CO_2_ER: FE(CH_4_) reaches 82.5% at −1.4 V in an H-cell and 84.9% at −1.2 V in a flow cell, with j_CH4_ up to 27.4 mA cm^−2^ (H-cell) and 365.1 mA cm^−2^ (flow), while CO is suppressed (≈1% beyond −1.4 V) and HER is minimized (FE(H_2_) ≈ 12.6% at −1.3 V); the FE(CH_4_) remains >81% over 12 h. Kinetic diagnostics attribute the rate/selectivity gains to a larger ECSA (C_dl_ = 2.72 mF cm^−2^) and lower charge-transfer resistance. Mechanistically, operando ATR-FTIR resolves the canonical *COOH → *CO → *CHO → *OCH_2_ → *OCH_3_ sequence to CH_4_, while DFT shows a narrowed band gap (0.179 eV) and a more positive d-band center at CuN_2_Cl_2_ that enhance charge transport and CO_2_ adsorption; notably, the dual-site catalyst shifts the RDS to *CO protonation at both sites with more favorable ΔG, and weakens *H adsorption, thereby suppressing HER. Together, these data establish dual-site programming within a crystalline COF as a powerful route to methane-selective CO_2_ electroreduction at technologically relevant current densities.

## 5. Summary and Outlook

Porphyrin-based COFs have achieved notable progress in both synthesis and application, yet substantial room remains for discovery. Their intrinsically ordered porosity and heteroatom-rich backbones endow stronger CO_2_ affinity and selectivity than many conventional semiconductors, positioning COFs as ideal platforms for CO_2_ reduction. A key next step is to translate COFs from “capture and conversion” models into broader synthetic paradigms—integrating CO_2_ reduction and C–C bond-forming (or insertion) chemistry to access higher-value fine chemicals.

Despite recent gains, porphyrin COFs still face challenges in synthetic complexity and performance. Rational control at the molecular—even electronic—level is needed to accelerate charge separation and transport while suppressing recombination, thereby improving solar-to-chemical energy conversion and catalytic turnover. Strategies may include strengthening through-bond/through-space conjugation, enhancing framework conductivity, engineering interlayer stacking and pore-surface functionality, and constructing robust linkages that balance crystallinity with stability and scalability.

From a photocatalytic standpoint, porphyrin-based COFs integrate strong visible-light absorption, tunable band gaps, and isolated (metallo)porphyrin M–N_4_ sites within an ordered porous scaffold, enabling efficient CO_2_-to-CO/formate conversion with competitive activity and selectivity. The examples summarized above show that optimizing in-plane conjugation, interlayer stacking, and heterostructure engineering is particularly important for enhancing light-driven CO_2_ reduction performance and will remain a key direction for future development of porphyrin COF photocatalysts.

In electrocatalysis, the predominant active motif is the M–N_4_ (metal–porphyrin) site, which often favors CO as the primary product; selective formation of higher-value hydrocarbons remains difficult. Deeper mechanistic insight—via operando spectroscopy, isotopic labeling, microkinetic analysis, and advanced computation—will be essential to map active-site states, identify rate-limiting steps, and guide the design of tandem/bimetallic sites, co-catalysts, and electrolyte environments that promote C–C coupling and controlled hydrogenation. With these advances, porphyrin COFs are poised to evolve into versatile and durable platforms for efficient CO_2_ utilization and value-added carbon manufacturing.

## Data Availability

No new data were created or analyzed in this study.

## Abbreviations

The following abbreviations are used in this manuscript:

COF	covalent organic framework
CO_2_RR	CO_2_ reduction reaction
fs-TAS	femtosecond transient absorption spectroscopy
PL	photoluminescence
TRPL	time-resolved photoluminescence
DRIFTS	diffuse reflectance infrared Fourier transform spectroscopy
DFT	density functional theory
PXRD	powder X-ray diffraction
AQE/AQY	apparent quantum efficiency/apparent quantum yield
